# Computational Drug Repurposing for Alzheimer’s Disease Using Risk Genes From GWAS and Single-Cell RNA Sequencing Studies

**DOI:** 10.3389/fphar.2021.617537

**Published:** 2021-06-30

**Authors:** Yun Xu, Jiming Kong, Pingzhao Hu

**Affiliations:** ^1^ Department of Biochemistry and Medical Genetics, University of Manitoba, Winnipeg, MB, Canada; ^2^ Department of Human Anatomy and Cell Science, University of Manitoba, Winnipeg, MB, Canada

**Keywords:** Alzheimer’s disease, computational approach, drug repurposing, gene signatures, genome-wide association study, pathway enrichment, single-cell sequencing study

## Abstract

**Background:** Traditional therapeutics targeting Alzheimer’s disease (AD)-related subpathologies have so far proved ineffective. Drug repurposing, a more effective strategy that aims to find new indications for existing drugs against other diseases, offers benefits in AD drug development. In this study, we aim to identify potential anti-AD agents through enrichment analysis of drug-induced transcriptional profiles of pathways based on AD-associated risk genes identified from genome-wide association analyses (GWAS) and single-cell transcriptomic studies.

**Methods:** We systematically constructed four gene lists (972 risk genes) from GWAS and single-cell transcriptomic studies and performed functional and genes overlap analyses in Enrichr tool. We then used a comprehensive drug repurposing tool Gene2Drug by combining drug-induced transcriptional responses with the associated pathways to compute candidate drugs from each gene list. Prioritized potential candidates (eight drugs) were further assessed with literature review.

**Results:** The genomic-based gene lists contain late-onset AD associated genes (BIN1, ABCA7, APOE, CLU, and PICALM) and clinical AD drug targets (TREM2, CD33, CHRNA2, PRSS8, ACE, TKT, APP, and GABRA1). Our analysis identified eight AD candidate drugs (ellipticine, alsterpaullone, tomelukast, ginkgolide A, chrysin, ouabain, sulindac sulfide and lorglumide), four of which (alsterpaullone, ginkgolide A, chrysin and ouabain) have shown repurposing potential for AD validated by their preclinical evidence and moderate toxicity profiles from literature. These support the value of pathway-based prioritization based on the disease risk genes from GWAS and scRNA-seq data analysis.

**Conclusion:** Our analysis strategy identified some potential drug candidates for AD. Although the drugs still need further experimental validation, the approach may be applied to repurpose drugs for other neurological disorders using their genomic information identified from large-scale genomic studies.

## Introduction

Alzheimer’s disease (AD) is the most common neurodegenerative diseases worldwide, which is characterized by progressive cognitive decline, including memory, speech, visuospatial performance and personality, leading to difficulties with basic daily activities (Weller and Budson, 2018). AD patients are not only at a higher risk of developing dementia but also more susceptible to medical comorbidities, such as osteoporosis, depression and cardiovascular diseases (Prince et al., 2013). The heterogeneity of AD has been illustrated by its complex pathobiology that might have been associated with genetic background, environmental factors and other causal triggers. So far, the majority of cases are late-onset AD, which occur after age 65, whereas the early-onset AD and autosomal-dominant AD constitute around 7% of all cases (Alzheimer Association, 2019). Within this frame, there is growing research around the neuropathological profile, thus hope to prevent or slow the rate of disease progression. Amyloid beta (Aβ) peptide, produced by the sequential cleavage of amyloid precursor protein (APP), was identified as the main constituent in amyloid deposition (Long and Holtzman, 2019). Several studies have suggested Aβ deposition may be required for the tau accumulation, unlike Aβ, the stage of tau pathology corresponds well with the phase of cognitive decline, which can serve as a predictor of AD progression (Nelson et al., 2012). Although it seems clear that the factors mentioned earlier are necessary but not sufficient to cause AD, so further clinical research is required to understand the underlying interplays (Long and Holtzman, 2019). Currently, degenerative disease like AD is incurable, and four Food and Drug Administration (FDA)-approved symptomatic medications including three cholinesterase inhibitors (donepezil, rivastigmine and galantamine) and memantine, an N-methyl-D-aspartate (NMDA) receptor modulator are available for the management of cognitive dysfunction in patients, but their overall efficacy is modest and not promising in the long term (Long and Holtzman, 2019), so an ongoing investigation is required to make any novel discoveries (Long and Holtzman, 2019).

The burden of drug development forced researchers to seek alternative approaches, drug repurposing, a strategy to find new uses for existing drugs by screening available compounds in the databases (Durães et al., 2018). A major advantage of drug repurposing is its safety, since the drug toxicity data are often available during clinical trials, and it can dramatically reduce the processing time (Zhang et al., 2016). Furthermore, drug repurposing makes use of large amount of genomic data accumulated in databases, thus to lower the investment of drug development. One recent in-silico study used a computational method to investigate ligand-protein interaction, thus exploring the potential antipsychotic drugs for AD (Kumar et al., 2017). Given the abundant resource of current biological research data and the expansion of computational algorithms, drug repurposing can advance drug development using robust and reliable data.

Recent large-scale genome-wide association analyses (GWAS) has led to the advancement of our understanding of the pathogenetic changes that trigger the development of AD (Tosto and Reitz, 2013). GWAS helps determine genes or gene networks that contribute to AD risk and highlights novel pathways for drug development (Tosto and Reitz, 2013). GWAS analyzes millions of single nucleotide polymorphisms (SNPs) across the genome to capture genetic risk variants from a disease prevalent in population (Bush and Moore, 2012). For the last decade, many GWAS studies have identified may susceptible genes which may be responsible for the altered risk of AD. On the other hand, the potential changes in cell composition and activity during neurodegeneration vary across cell types, suggesting cell-type-specific gene expression changes have potential to be associated with AD pathology (Mathys et al., 2019). It is difficult to identify the developmental features of major cell types that underlie diseases’ cellular basis. Single-cell sequencing (scRNA-seq) can study complex cellular changes in disease by quantifying gene expression at the level of individual cells. ScRNA-seq analyzes single-cell transcriptomes from different cortical regions to identify distinct disease-associated subpopulations (Zhong et al., 2018). Thus, scRNA-seq can link transcriptional alterations associated with AD to cell-type specific gene expression changes. Several studies have analyzed transcriptome profiles of single cells in various regions of cerebral cortex to dissect the cellular basis behind the neurodevelopmental disorders (Lake et al., 2016; Zhong et al., 2018). Therefore, using single-cell transcriptomes to identify AD -associated risk genes for searching for drug targets may be potentially more effective, by targeting the major cell-type-specific genes involved in AD progression.

The enormous potential of drug repurposing using computational methods motivated us to identify potential anti-AD agents through enrichment analysis of drug-induced transcriptional profiles of pathways using AD-associated risk genes identified from genome-wide association analyses (GWAS) and single-cell transcriptomic studies. We applied the computational tool “Gene2Drug” previously developed by Napolitano et al. (2017) on the genomic data from GWAS and single-cell transcriptomic studies, and analyzed through gene expression profiles of five cell lines treated with 1,309 different small molecules from the Connectivity Map (CMap). We hypothesize that searching for drugs that could induce transcriptional responses to the pathways involving AD risk genes can accelerate the identification of suitable drug candidates. Our findings further purport the opportunities for using AD risk genes for drug discovery and repurposing.

## Materials and Methods

**FIGURE 1:**
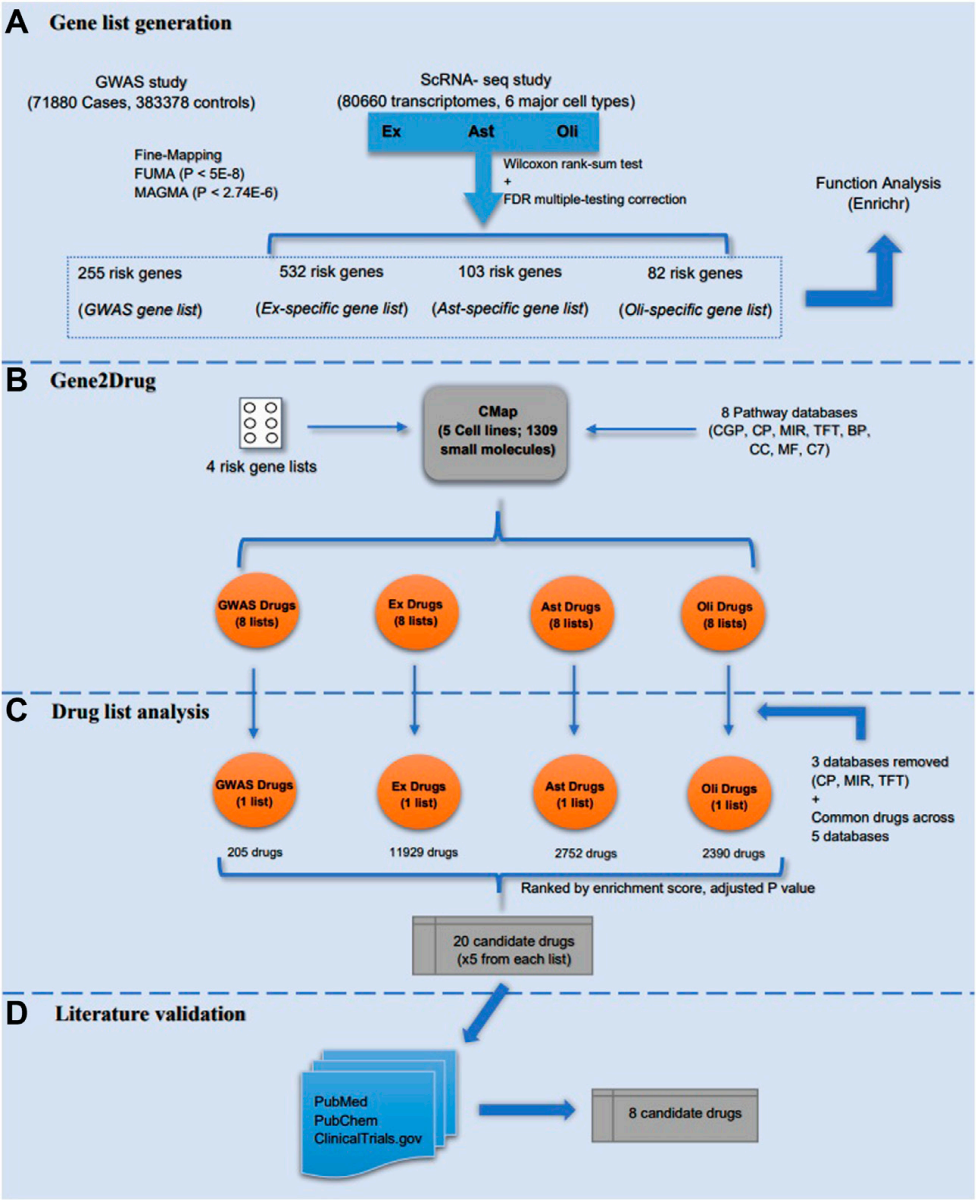
Study design. The study included four major steps: **(A)** four risk genes lists (GWAS, Ex, Ast, and Oli) were generated from GWAS and scRNA-seq studies, respectively. Meanwhile, a preliminary functional analysis of the gene lists was performed in Enrichr; **(B)** Gene2drug analysis was then performed on the four risk gene lists by incorporating eight pathways databases; **(C)** drug lists were analyzed and filtered based on enrichment score and adjusted *p*-value to select top five drugs from each list; **(D)** the identified AD candidate drugs were validated through literature review to determine the most promising candidates.

Our analysis strategy to repurpose drugs for AD using AD risk gene lists obtained from GWAS and scRNA-seq studies consists of four main steps (Figure 1): generation of risk gene lists from two recent large-scale studies, identification of AD candidate drugs from risk gene targets using Gene2Drug algorithm and pharmacogenetic profiles, drug lists analysis for all four gene lists and validating the identified AD drugs through literature review. Each step is illustrated in detail below.

### Data Sources

We obtained genes strongly associated with AD and generated four gene lists using two large-scale genomic studies.

#### GWAS-Based AD Risk Gene List

Jansen et al. (2019) performed a SNP-based GWAS study of diagnosed late-onset AD (71,800 cases, 383,378 controls), which is the largest GWAS study of diagnosed late-onset AD. Three approaches were used to identify risk genes related to the AD-associated SNPs. First, 136 genes were selected from the Supplementary Table S8 of (Jansen et al., 2019), which were identified from the fine mapping of the sets of risk SNPs; Second, 192 mapped genes were implicated in Functional Mapping and Annotation (FUMA) (positional mapping, eQTL, chromatin interaction mapping; SNPs with *p* < 5 × 10^−8^) [Supplementary Table S13 of (Jansen et al., 2019)]; Third, 97 known protein-coding genes were significantly associated with AD identified using Multi-marker Analysis of GenoMic Annotation (MAGMA) (*p* < 2.74 × 10^−6^) from the GWAS study [Supplementary Table S18 of (Jansen et al., 2019)]. These genes are collectively included to form a list of 255 unique late-onset AD associated risk genes (Supplementary Table S1A).

#### ScRNA-Seq Based Cell-Type Specific AD Risk Gene Lists

Mathys et al. (2019) analyzed 80,660 single-nucleus transcriptomes from the prefrontal cortex of 48 individuals with varying degrees of Alzheimer’s disease pathology, and they characterized cell-type specific gene expression changes for all six major brain cell types [excitatory neurons (Ex), inhibitory neurons (In), astrocytes (Ast), oligodendrocytes (Oli), oligodendrocyte progenitor cells (Opc), and microglia (Mic)]. This is the largest scRNA-seq analysis of AD. Notice that most significantly altered genes for each cell type occurred in early-vs. late-pathology as opposed to no-versus AD and no-versus early stage. A fraction of upregulated genes found by comparing early and late pathology is involved in protein folding, autophagy, apoptosis, and stress response (Mathys et al., 2019). Mathys et al. made a global consistent comparison analysis between scRNA-seq and bulk RNA-seq differential expression analysis, and showed that Ex, Ast and Oli have significant consistency (Bonferroni adjusted *p*-value<0.05) while In, Opc and Mic have no significant consistency (Bonferroni adjusted *p*-value>0.05) [please refer to Figure 1F from (Mathys et al., 2019)]. Hence, we focused our analysis on differentially expressed risk genes identified from Ex, Ast and Oli by comparing early and late pathology. To select the differentially expressed AD risk genes generated by Mathys et al. (2019) [Please refer to Supplementary Table S2 (Mathys et al., 2019)], we applied two cutoffs to the differential genes identified from each of the three cell types (Ex, Ast and Oli): 1) each gene should have an adjusted *p*-value<0.05; and 2) each gene should also have a fold change at least 1.5. As a result, we selected 717 significantly differentially expressed genes, which included 532, 103, and 82 differentially expressed genes from Ex, Ast, and Oli subpopulations, respectively (Figure 1). Finally, three lists of cell-type specific AD risk genes and one list of GWAS late-onset AD risk genes were each inputted in turn for computational analysis (Supplementary Table S1).

### Gene Functional Analysis and Gene Overlap

As a preliminary assessment, each of the four gene lists were analyzed using Enrichr software (Kuleshov et al., 2016) to search for potential involvement of biological pathways and molecular function (Supplementary Tables S2A–D). We then looked at the association between the gene lists and the target genes of the current AD candidate drugs in phase 1, 2, and 3 from Cummings et al. (2019), a study on the discussion of pharmacologic agents in clinical trials for the treatment of AD, therefore, to reveal possible gene overlaps. The target genes of the drugs from the three phases of clinical trials were manually retrieved from Therapeutic Target Database (TTD) (Chen, 2002), DrugBank (Wishart, 2006) and PubChem databases (Kim et al., 2015). To manually search for these target genes, drug synonyms and mechanisms of actions were used to assist in finding drug targeted genes for each of the three phases of clinical trials. (Supplementary Tables S2E–H). The gene overlaps were then analyzed using Enrichr GO Biological Process 2018 and Enrichr GWAS Catalog 2019 databases.

### Computational Analysis of the AD Risk Genes for Drug Repositioning

Gene2Drug. Once the AD-associated risk genes are curated from the two recently published large-scale genomic studies, Jasen et al. (Jansen et al., 2019) and Mathys et al. (Mathys et al., 2019), candidate drugs that significantly target these genes can potentially be explored. A novel approach developed by Napolitano et al. (2017) aims to advance drug repositioning by combining drug-induced transcriptional responses with the associated pathways. This unique method enables the identification of drugs that induce significant transcription of pathways that involve the target genes as we showed before (Grenier and Hu, 2019). The authors implemented the method as an online tool named “Gene2Drug” (Grenier and Hu, 2019), and an R package “gep2pep” was developed for the tool to analyze in R studio (Napolitano et al., 2019). Although the use of tool “Gene2Drug” may help prioritize drugs directly acting on the therapeutic target, any drug directly or indirectly modulating the expression of the target-related pathways will be selected as a potential candidate for drug repurposing (Grenier and Hu, 2019). In order to identify drugs that modulate the target genes of interest, the “Gene2Drug” tool exploits the gene expression data from the Connectivity Map (CMap), including gene expression profiles of five cell lines treated with 1,309 different small molecules (Lamb, 2006). For each target gene in a gene set of interest, Gene2Drug will automatically generate a subset of pathways (one for each drug) containing the input gene, then apply Gene Set Enrichment Analysis (GSEA) to compute an enrichment score and a *p*-value for each drug (Subramanian et al., 2005). The final output contains a list of drugs ranked by the corresponding *p*-values, and pathways that tend to be transcriptionally upregulated (downregulated) are at the top (bottom) of the results. In other words, the top-ranked drugs are more likely to activate the therapeutic target comparing with the bottom-ranked drugs (Napolitano et al., 2017).

Pathway analysis. For our purposes, we performed analysis individually on the AD risk genes derived from the GWAS study and the cell-type-specific differentially expressed genes that correspond to the early-vs. late-stage AD group, to determine if the given AD risk genes are significantly enriched in the drug-treated pathways. The use of GSEA as part of the data preprocessing phase, through the implementation of gep2pep R package, supports the conversion of gene expression profiles to pathway expression profiles; thus pathway-based analytic tools including the drug set enrichment analysis (DSEA) and Gene2Drug can be applied (Napolitano et al., 2019). Instead of identifying pathways that are targeted by a set of drugs, pathway set enrichment analysis (PathSEA) procedure (Napolitano et al., 2019) performs on computed pathway expression profiles to yield AD candidate drugs that induce significant transcriptional responses to the pathways involving the risk genes, thus, making the Gene2Drug approach more comprehensive than traditional computational analysis. Table 1 lists the eight pathway databases used for the Gene2Drug analysis on AD risk genes. Multiple pathway databases were used to ensure that the prioritized drugs target as many pathways as possible that the AD risk genes were involved in. Our Gene2Drug analysis took one AD risk gene as input each time and found all of the pathways that the gene is involved in by using the eight reference databases. Similarly, enrichment scores and *p*-values were calculated for all the CMap drugs by means of PathSEA, and the final output was ranked by corresponding *p*-values to assess how much the drugs modulate these pathways. The enrichment score is a value ranges from-1 to 1 that represents how much the drug treatment regulates the pathway, and it is calculated using a generalization of the Kolmogorov- Smirnov statistic (Napolitano et al., 2017). Computed *p*-values were further adjusted using the Benjamin-Hochberg procedure to account for the high false-positive rate associated with multiple testing procedures. One pathway-drug matrix was generated by applying the method to one pathway database (each gene list passes through all eight pathway databases); thus, eight pathway-drug matrices were obtained for each of the GWAS and cell-type-specific gene lists. Overall, a total of thirty-two drug lists have been generated for all four gene lists by applying the pathway analysis procedure. For a drug to be considered for the second stage of filtering and selection, it had to meet the false discovery rate cut-off of 0.05; thus all thirty-two drug lists (eight drug lists for each of the four gene lists) were initially filtered after applying the cut-off adjusted *p*-value. These drug lists were then further analyzed to prioritize potential drugs.

**TABLE 1:** Pathway databases used for Gene2Drug analysis.

Source	Name	Description
MSigDB	CGP	Genetic and chemical perturbations
MSigDB	CP	Expert-defined canonical pathways
MSigDB	MIR	microRNA targets
MSigDB	TFT	Transcription factor targets
MSigDB	GO BP	Gene ontology–Biological processes
MSigDB	GO CC	Gene ontology–Cellular component
MSigDB	GO MF	Gene ontology–Molecular function
MSigDB	C7	Immunologic signatures

The gep2pep package was used to perform this analysis (Napolitano et al., 2019). Default parameters were used when running this package.

### Ranking and Selecting Significant Drugs for Cell-Type Specific and GWAS Risk Genes

For the remaining candidate drugs that passed the adjusted *p*-value cut-off, we obtained thirty-two drug lists as outputs of the four gene lists. Notably, the expert-defined canonical pathways (CP), microRNA targets (MIR), transcription factor targets (TFT) databases generated the fewest candidate drugs, and they were neglected to maximize the number of common drugs found across the remaining five drug lists. To this end, a total of twenty drug lists would be used for the next stage of selection. First, an adjusted *p*-value rank column was added for each of the twenty drug lists by assigning lower ranks to smaller adjusted *p*-values. Meanwhile, an enrichment score rank column was added to each of the twenty tables by assigning lower ranks to higher enrichment scores. Secondly, the four lists of common drugs found across the databases would have five different adjusted *p*-value rank and five enrichment score rank depending on the database. Therefore, the median adjusted *p*-value rank and median enrichment score rank were then calculated and added to each common drug list. Finally, the four lists of common drugs associated with each of the gene lists were sorted by median enrichment score rank (from smallest to largest) and then by median adjusted *p*-value rank (from smallest to largest).

### Literature Validation of the Identified AD Candidate Drugs

Once the most relevant and significant drugs for each gene list are ranked at the top (lower rank), a literature review was performed to validate the top five drugs for each gene list (a total of twenty drugs). The purpose of validation was to ensure that the drug produces the therapeutic effects intended in previous studies done on the particular drug. On the other hand, it is crucial to find out any undesired side effects during clinical trials. Validation was performed using PubMed (Lindsey and Olin, 2013), PubChem (Kim et al., 2015) and ClinicalTrials.gov (Zarin et al., 2011). Studies searched from PubMed regarding AD were used to support the relative efficacy of drugs. PubChem provides information related to the toxicity of the drugs to see if they are safe to use. The AD-related data from ClinicalTrials.gov includes all the drugs at different stages of clinical trials, and the tool can help see if the prioritized drugs have been tested or approved.

## Results

### Function Enrichment Analysis of the AD Risk Genes

The functions of each of the four gene lists (Supplementary Table S1) were analyzed using Enrichr software to search for potentially enriched biological pathways (Supplementary Tables S2A–D). Several genes (BIN1, ABCA7, APOE, CLU, SORL1, and PICALM) in the GWAS gene list are negative regulators of APP catabolism suggests a downregulation of APP breakdown. The majority of filtered Ex genes demonstrated essential roles in fundamental cellular activities, while some genes are significantly enriched in the regulation of microtubules, fibroblasts and immune cells (adjusted *p*-value<0.05) which have been previously suggested in AD research. A similar observation was obtained for filtered Oli genes, but interestingly, Enrichr analysis revealed that several filtered Ast genes were shown to be directly correlated with myelin maintenance (CXCR4, SOD1) and axonogenesis (NRP1, FEZ1, S100A6, CXCR4, and CCK) (adjusted *p*-value<0.05) (Supplementary Table S1G). Enrichr Pathway analysis on the generated target genes from Cummings et al. (2019) consists of more diverse pathways, including neurotransmission, immune response, and cellular homeostasis. Therefore, the results confirmed that the current AD candidate drugs are also involved in other physiological pathways.

To confirm whether any drugs have targeted these curated AD-associated risk genes in Phase 1, 2 or 3 clinical trials for AD (called clinical AD drug targets, which include 34, 109, and 49 genes for Phases 1, 2, and 3 trials, respectively) (Cummings et al., 2019), we searched for gene overlaps between the GWAS gene list and the clinical AD drug targets, which produced five gene matches (TREM2, CD33, CHRNA2, PRSS8, and ACE) (Supplementary Table S2I). These genes are significantly involved in protein phosphorylation, stem cell differentiation, immune response and blood pressure, based on Enrichr Ontology analysis (adjusted *p*-value<0.05). As for the Enrichr Disease results, the fives genes have implications in microvascular complications of diabetes, as well as late-onset AD (Supplementary Table S2J). The overlap analysis between the filtered Ex gene list and the clinical AD drug targets produced three gene matches (TKT, APP, and GABRA1), which mainly plays a role in regulating microglial cell activation, astrocyte activation and neuron maintenance, suggesting important pathway in AD development (Supplementary Table S2H). However, no gene overlap was found for Oli and Ast cell types. The analysis suggests that other drugs targeted these AD-associated risk genes can potentially be repurposed for AD. The Enrichr Disease analysis also revealed that these overlapped genes have a significant association with generalized epilepsy besides AD (adjusted *p*-value<0.05).

### Prioritize Top Significant AD Candidate Drugs

For the twenty drugs prioritized from Gene2Drug analysis, ones (12 of the 20) that are duplicated or lacked sufficient information based on the searches in PubMed were excluded from the analysis. Several top-ranked drugs (ellipticine, alsterpaullone, and tomelukast) obtained Ex risk genes were also observed for Oil or Ast, but no drug overlap was seen between the cell-type-specific drugs and the GWAS drugs. The eight identified AD candidate drugs are listed and summarized in Tables 2, Tables 3 with their literature evidence on toxicity profiles, targeted AD pathologies, drug targets, clinical trials. Among the eight candidate drugs, four of the drugs (alsterpaullone, ginkgolide A, chrysin and ouabain) have shown repurposing potential for AD based on their preclinical evidence and moderate toxicity profiles. These drugs are required to be studied in a clinical setting to clarify their long-term safety. For the remaining drugs (ellipticine, tomelukast, sulindac sulfide and lorglumide), they are unsuitable for further investigations either due to severe adverse effects or lack of multi-prolonged mechanism of actions. We also searched for the FDA-approved drugs including donepezil, rivastigmine, galantamine and memantine in the finalized four drug lists. Memantine is present in GWAS, Ex and Oli gene lists, and galantamine was only found in Ex gene list. Due to the massive candidate drugs computed from the approach, these two drugs were not ranked at the top but they are still considered statistically significant.

**TABLE 2:** Summary of literature evidence for prioritized drugs obtained from Gene2Drug analysis.

Drugs	Gene list	Summary of evidence (drug targets)	Toxicity
Ellipticine	Excitatory neuron	• Inhibition of proinflammatory cytokines (TNF-α, IL-6), oxidative stress, Aβ production, early apoptosis signal by direct suppression of JNK-AP-1 pathway Krilleke et al. (2003); Tian et al. (2019)	Mild side effects such as nausea, vomiting, hypertension, muscular cramp. O'sullivan et al. (2013)
	Oligodendrocyte		
Alsterpaullone	Excitatory neuron	• Reduction of cytoskeletal abnormalities, neuronal death, tau hyperphosphorylation, Aβ formation by inhibiting GSK-3β and CDK5. Leost et al. (2000); Phiel et al. (2003); Shrestha et al. (2013); Maqbool et al. (2016)	May have mild side effects such as diarrhea, nausea, vomiting. Senderowicz (2000)
	Astrocyte	• May promote hippocampal neurogenesis, proliferation and differentiation Maqbool et al. (2016)	
		• To a lesser extent: Additional effects on limiting early toxic protein deposition and glial cell-mediated neuroinflammation regulated by ERK1/2. Inoue et al. (2012); Sun and Nan (2017)	
Tomelukast	Excitatory neuron	• Inhibition of CysLT1 involved in neuroinflammation, cell apoptosis, disrupted BBB and vasculature, disrupted learning and memory Hoover (1990); Michael et al. (2019)	Damage to liver; gastrointestinal: Hypermotility, diarrhea Hoover (1990) Hagopian (1988)
	Oligodendrocyte	• Increasing anti-inflammatory cytokines and neuroprotective molecules (HSP70, IkBα, and IkBβ) by activating PPAR-γ Feinstein (2004)	
Ginkgolide A	Astrocyte	• Modulating post-translational modifications of α-tubulin to preserve microtubule dynamics. Zhang et al. (2015); Kawamura et al. (2016)	Mild side effects such as headache, nausea, vomiting, allergic skin reactions. Gornik and Creager (2013)
		• Inhibitor of NMDA receptor involved in impaired neurotransmission and cognitive decline Kuo et al. (2018)	
		• Reduction of phosphorylated tau proteins by activating PI3K-Akt pathway to phosphorylate GSK3β at ser9. Dudek et al. (1997); Chen et al. (2012)	
Chrysin	Oligodendrocyte	• Reduction of neurotrophic factors (NO, TNF-α) by downregulating NF-kB p65 and C/EBPs Gresa-Arribas et al. (2010)	Chrysin has been shown to induce toxicity in trout liver cells Siddiqui et al. (2018)
		• Improvement of memory function in the hippocampus caused by Aβ deposition Vedagiri and Thangarajan (2016)	
		• Upregulation of antioxidative and cytoprotective genes (HO-1, CAT and SOD) by activating Nrf2 under oxidative stress Angelopoulou et al. (2020)	
		• Promotion of cell survival and inhibition of mitochondrial dysfunction and autophagy dysregulation through activation of MEF2D Angelopoulou et al. (2020)	
Ouabain	GWAS	• Reduction of pro-inflammatory cytokines by inhibiting Na^+^/K^+^ - ATPase involved in upregulating NF-kB and NLRP3. de Lores Arnaiz and Ordieres (2014)	Mild side effects: Nausea, vomiting, pulse irregularities
		• Enhancement of TFEB in which to increase APP and tau degradation. Xiao et al. (2015)	
Sulindac sulfide	Excitatory neuron	• May slow neuronal aging, memory deficits and prevent early accumulation of Aβ oligomers induced by COX. Lee et al. (2008); Woodling et al. (2016)	Gastrointestinal effect including ulceration, bleeding and perforation Park (1982)
		• Preferential inhibition of γ42-secretase to decrease Aβ42 aggregation Takahashi et al. (2003)	
		• Reduction of proinflammatory cytokines and APP aggregation by augmenting PPAR-γ Khan et al. (2019)	
Lorglumide	Oligodendrocyte	• Reduction of dopamine neurotransmission by regulating CCK_A_ receptor. Gonzalez-Puga et al. (2005); Ballaz, 2017)	Mild side effects Makovec et al. (1987)

**TABLE 3:** Clinical trial/Preclinical research summary for AD candidate drugs.

Drugs	Possible targeted AD pathology	Other diseases tested for (with derivatives)	Remaining work required
Ellipticine	• Proinflammatory response	Elliptinium acetate	• Need more clarification of mechanism of action and direct effect on reducing neuronal cell death
	• Aβ overproduction	Breast cancer (Phase I/II) O'sullivan et al. (2013)	• Clinical trial required to confirm preclinical evidence in patients with AD.
	• Early apoptotic signal	Retinal carcinomas (Phase II)	
	• Oxidative stress	Datelliptium	
		Lymphomas (Phase I)	
Alsterpaullone	• Aβ formation from APP	Preclinical study for group 3 medulloblastomas Faria et al. (2015)	• Clinical work required to identify effect on AD pathology in animal and human
	• Tau pathology		• Clarification of optimal dosage and related side effects
	• Neurotoxicity		• Evidence of safety with long-term use
	• Cytoskeletal abnormalities		
	• Glial cell-induced neuroinflammation		
	• Cognitive decline		
Tomelukast	• Microglia-induced neuroinflammation	Asthma (withdrawal) Reques and Rodriguez (1999)	• Severe adverse effects need to be limited using lower dosage in clinical work
	• Impaired glutamatergic neurotransmission		• Preclinical study in patients with AD required to support mechanism of action
	• Impaired neurogenesis		
	• Disrupted BBB and vasculature		
	• Cognitive decline		
Ginkgolide A	• Microtubule reduction	Intravenous alteplase thrombolysis (Recruiting) ClinicalTrials.gov (2018)	• Evidence of safety with long-term use
	• Impaired synaptic transmission	Intrauterine growth restriction [approved] ClinicalTrials.gov (2015)	• Clinical evidence required to clarify effect on cognitive improvement and early prevention in AD
	• Early apoptosis		• Further clinical work in patients with AD
	• Tau phosphorylation		
	• Cognitive decline		
Chrysin	• Aβ overproduction	Under basic research for inflammation and neurological disorders Rashid and Sultana (2015)	• Evidence of safety with long-term use
	• Inflammation		• Preclinical evidence required to clarify effect on cognitive improvement and mechanism of action
	• Neurotrophic factors		• Further clinical work in patients with AD
	• Oxidative stress		
	• Mitochondrial dysfunction		
	• Autophagy dysregulation		
Ouabain	• Toxic tau aggregation	Not directly involved in clinical trials	• Further *in vivo* or *in vitro* work required to understand mechanism of action on AD pathology
	• Neuroinflammation		• Clinical evidence
	• Cognitive impairment		• Evidence of safety with long-term use
Sulindac sulfide	• Aβ formation from APP	Not directly involved in clinical trials	• More clinical and/or epidemiological evidence needed
	• Memory impairment		• Clarification of optimal dosage for adverse effects
	• Oxidative stress		• Further *in vivo* or *in vitro* work required to understand mechanism of action on AD pathology
	• Proinflammatory cytokines		• Clinical evidence
Lorglumide	• Impaired neurotransmission	Basic research for gastrointestinal diseases, and some forms of cancer Herranz (2003); Berna et al. (2007)	• Controversial mechanisms of action on AD need to be clarified
	• Metabolic and cardiovascular risk factors		• Clinical evidence
			• Evidence of safety with long-term use

### Literature Evidences for Mechanism of Actions of the Predicted Drugs

**FIGURE 2:**
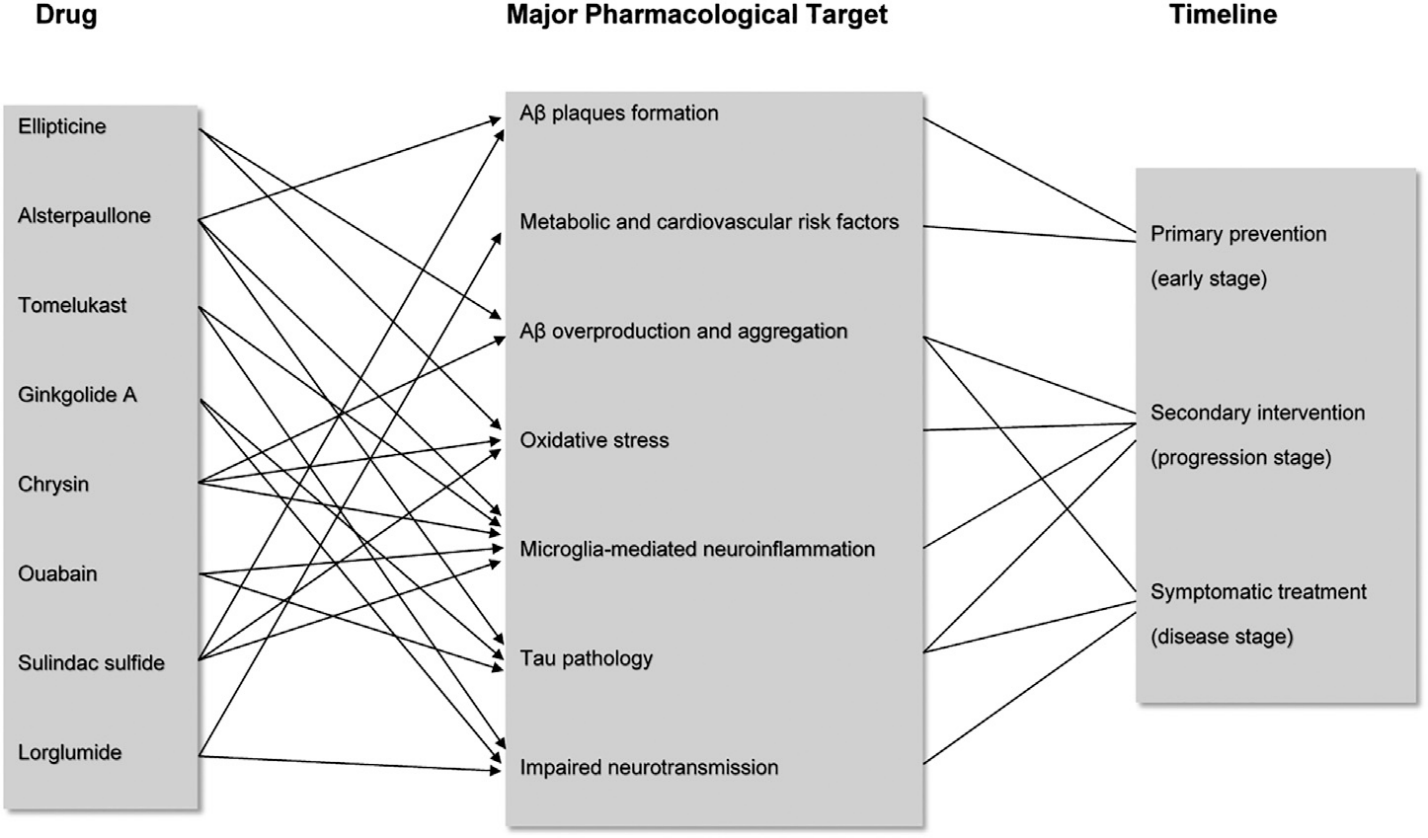
Major pharmacological targets of the eight candidate drugs during AD progression. Candidate drugs are each associated with several major targets involved in different timepoints of AD progression. Arrows and lines show the correlation between drug and pharmacological targets based on the literature evidence, as well as the correlation between targets and disease progression timeline.

For the eight prioritized candidate drugs identified from the drugs lists using the Gene2Drug algorithm, their overall pharmacological targets are characterized by different stages of AD progression (Figure 2). It is necessary to understand the mechanism of actions of these drugs for better AD prevention and treatment. AD is characterized by complex pathologies, and it is believed that some drugs are involved in several regulatory pathways, which may pose an advantage for multi-targeted therapies. The potential literature evidences for mechanism of actions of the eight predicted candidate drugs are illustrated below.

#### Ellipticine

Ellipticine is a potent antineoplastic compound that has shown efficacies against several types of cancers. The drug has appealing clinical potential due to its limited toxic side effects and its complete lack of hematological toxicity (Stiborova et al., 2006). The antitumor function of ellipticine was suggested to be mediated by inhibition of DNA topoisomerase II activity and oxidation of cytochrome P450 (CYP). The drug was primarily investigated for use in cancer treatment due to its participation in cell cycle arrest and induction of apoptosis (Tian et al., 2019). It is currently thought the drug exerts protective effects against inflammatory responses through suppression of c-Jun N-terminal kinase (JNK)- activator protein-1 (AP-1) signaling pathway, which is involved in gene regulations, such as growth factors, cytokines and apoptosis. Among the pro-inflammatory factors, the secretion of tumor necrosis factor (TNF-α) and interleukin-6 (IL6) by activated macrophages were downregulated by ellipticine (Tian et al., 2019). The link between the JNK signaling pathway and ellipticine could make it possible to moderate AD symptoms. It has been suggested that JNK3 plays a role in enhancing Aβ production, and development of neurofibrillary tangles, which in turn results in neuroinflammation and neurodegeneration. Moreover, JNK has also been shown to activate apoptosis-related protein directly and further induce downstream caspases. Studies have suggested that the increased risk of neurodegeneration in the early age of AD patients could be the result of apoptotic cell signaling and oxidative stress, mediated by JNK activation (Krilleke et al., 2003; Tian et al., 2019). Thus, interfering with JNK pathways using inhibitors appears to be a potential strategy to prevent early neuronal death in AD. Although ellipticine was able to inhibit the JNK-AP-1 signaling pathway, extracellular signal-regulated kinase/nuclear factor-kB (ERK/NF-kB) pathway was not affected. NF-kB transcription factors are important in coordinating immune and inflammatory responses where they can control programmed cell death by engaging crosstalk with the JNK signaling pathway (Papa et al., 2004). Therefore, inhibition of the JNK cascade to control TNF-σ-induced apoptosis critically depends on NF-kB. This illustrates that NF-kB blockers may have better potential in treating excessive cell death in AD, whereas ellipticine could only diminish early but not late cellular apoptosis associated with AD. Currently, derivatives of ellipticine have been clinically tested for various forms of cancers, and they are often used for the treatment of obesity and certain genetic diseases such as parkinsonism and neuropathy (O'sullivan et al., 2013).

#### Alsterpaullone

Alsterpaullone is a small-molecule inhibitor that targets cyclin-dependent kinases (CDK), where it induces cell cycle arrest and promotes apoptosis of tumor cells. The apoptosis-inducing effect of the drug in tumorigenesis is found to be mediated *via* the mitogen-activated protein kinase (MAPK) signaling pathway (Yin et al., 2018). Although alsterpaullone is primarily concerned with the anti-cancer treatment, it also targets relevant human protein kinases relevant to AD.

It has been found that a range of protein kinases contributes to the progression of AD by phosphorylating tau *in vitro*, among the physiologically relevant proteins, glycogen synthase kinase-3β (GSK-3β) appears to be one of the targets of alsterpaullone (Leost et al., 2000). Ubiquitously expressed at high levels in the brain, GSK3 has been implicated in neuronal functions by being involved in the WNT signaling pathway, axonal outgrowth, and neuronal polarization. As an isozyme of GSK3, dysregulation of GSK-3β gives rise to many lethal diseases, including neurodegenerative disorders such as AD (Maqbool et al., 2016). GSK-3β is found highly activated in granulovacuolar degenerated neurons wherein it hyperphosphorylates the microtubule-associated tau proteins, resulting in the fatal accumulation of neurofibrillary tangles which will weaken neuronal synapses (Maqbool et al., 2016). On the other hand, GSK-3β also mediates Aβ production from the precursor proteins and results in neurotoxicity (Phiel et al., 2003). Various GSK3 inhibitors have been reported to reduce the amount of Aβ as well as the tau hyperphosphorylation in both neuronal and nonneuronal cells. More importantly, the efficacy of these inhibitors was shown in promoting hippocampal neurogenesis, nerve cell proliferation, migration and differentiation (Maqbool et al., 2016). This illustrates why the enzyme should be viewed as a target for AD therapy, and alsterpaullone, as an ATP competitive inhibitor, work to inhibit GSK-3β activation.

Another AD-associated protein kinase targeted by alsterpaullone is neuronal cyclin-dependent kinase 5 (CDK5) p25 (Leost et al., 2000). CDKs generally are involved in cell cycle regulation, transcription, neuronal functions and apoptosis, particularly, CDK5/p25 activation was indicated to induce cytoskeletal abnormalities and neuronal death in AD patients (Shrestha et al., 2013; Shah and Lahiri, 2014). As an important modulator of neuronal activity, CDK5/p25 was also found to potentiate the phosphorylation of tau by GSK-3β by colocalizing on the AD-specific sites. Several synthesized CDK5/p25 inhibitors have been used to prevent neuronal cell loss and exert a neuroprotective effect (Leost et al., 2000). Although it appears that alsterpaullone has a higher sensitivity to GSK-3β than CDK5/p25, the compound still showed a great impact on tau pathologies. To this extent, the inhibitory effect of alsterpaullone on both protein kinases further reinforces its candidacy for drug repurposing.

**FIGURE 3:**
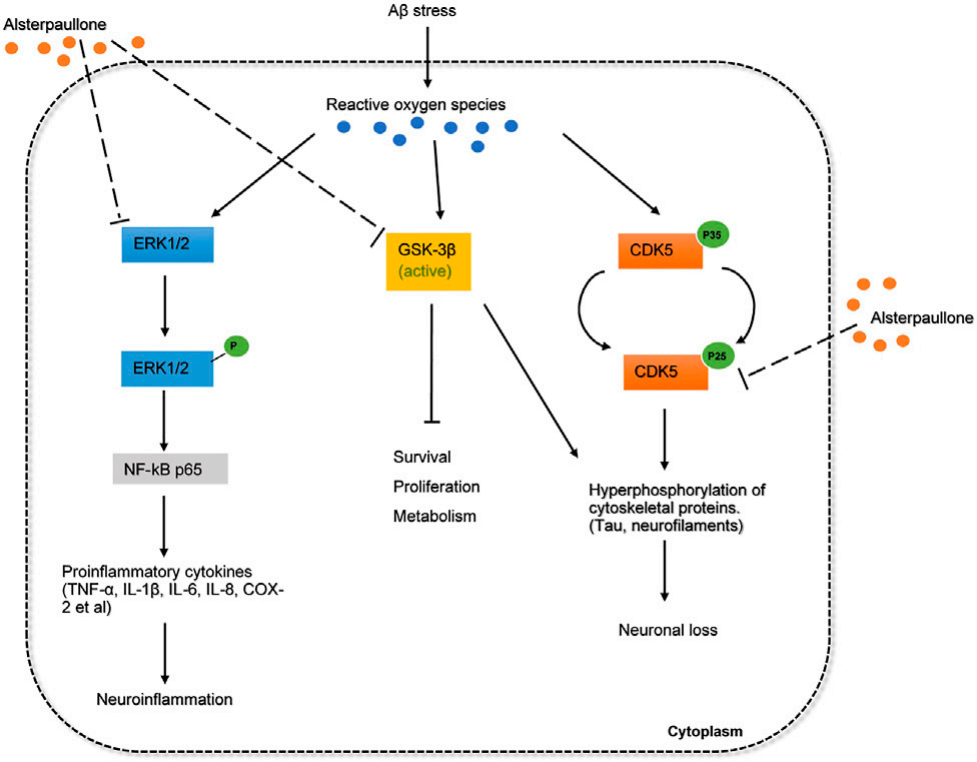
The inferred mechanism of alterpaullone in AD pathology. The drug acts through the inhibition of GSK3β, CDK5, and ERK1/2. The inhibition of GSK3β and CDK5 leads to reduced hyperphosphorylation of toxic cytoskeletal proteins and promote cell survival and proliferation. The inhibition of ERK1/2 leads to deactivation of NF-kB p65 that produces proinflammatory cytokines during inflammation. The solid dash lines show the interactions of protein kinases and signaling cascades. The long-dashed lines show the pharmacological action of drug.

In addition to GSK-3β and CDK5, to a lesser extent, alsterpaullone also targets ERK1/2 cascade to suppress tau phosphorylation (Inoue et al., 2012). ERK1/2 cascade is generally activated by a variety of upstream kinases such as MAPK 1/2, and the abundance of ERK1/2 in the adult brain allows it to regulate a range of processes from synaptic plasticity, inflammation, memory formation to cell survival and death (Sun and Nan, 2017). Although ERK1/2 activation has been associated with synaptic plasticity, brain cell differentiation and proliferation, a number of studies have suggested that ERK1/2 cascade can promote neuronal damage in several neuronal systems. *In-vitro* studies have demonstrated that ERK1/2 signaling pathways contribute to the inflammatory response in microglia and astrocytes (Sun and Nan, 2017). Furthermore, activated ERK1/2 is especially found in intracellular NFTs located in the subpopulation of neurons with early AD-related protein deposition. The deregulation of the ERK1/2 cascade may impair hippocampal function and memory formation (Sun and Nan, 2017). Thus, ERK1/2 pathways may represent a valid therapeutic target in drug repositioning for AD. The overall mechanism of alsterpaullone involved in AD pathology is proposed and depicted in Figure 3. If alsterpaullone could effectively block the phosphorylation of ERK1/2 by the upstream kinases, it will be considered an attractive drug for preventing neuronal death in AD.

#### Tomelukast

Tomelukast is an orally active cysteinyl leukotriene type 1 receptors (CysLT1) antagonist commonly investigated to treat asthma and related respiratory disorders (Hoover, 1990). Cysteinyl leukotrienes (CysLT) are inflammatory lipid mediators synthesized by various cells, in which it induces human allergic and hypersensitive reactions. Therefore, antagonism of cysteinyl leukotrienes activity by blocking it from binding to receptors with tomelukast is an often-used approach (Hoover, 1990). Moreover, the leukotriene system affects various cell types from smooth muscle cells, epithelial cells to hippocampal neurons in the adult brain. It has been found that the leukotriene system is involved in neuroinflammation, either directly or indirectly by influencing astrocytes and microglia. Abnormal expression of glial cells such as microglia-expressed genes in phagocytosis has been shown relevant for AD pathogenesis (Michael et al., 2019). In healthy individuals, the microglia are triggered to participate in Aβ plaque clearance, but more proinflammatory cytokines will be produced as microglia becomes dysfunctional during AD pathology. Aside from microglia, astrocytes regulate brain homeostasis by releasing various neurotransmitters (Michael et al., 2019). In AD conditions, Aβ plaque-associated astrocytes will result in deregulated transmission at glutamatergic synapses, which contributes to cognitive decline. Thus, the proinflammatory effect of CysLTs on glial cells needs to be inhibited at the receptor level to positively affect neuroinflammation (Michael et al., 2019). In the neurogenesis context, leukotrienes are thought to adversely affect neurogenesis, since the inhibition of leukotriene was shown to promote the proliferation of neural progenitors and restore hippocampal neurogenesis in adult rats (Michael et al., 2019). AD is also associated with pathological hallmarks related to the blood-brain barrier (BBB), and CysLTs could increase BBB permeability and results in leakage at the capillary level (Michael et al., 2019). The pleiotropic effects of CysLTs on various aspects of AD pathology such as neuronal apoptosis, neuroinflammation, disrupted BBB and vasculature, could be simultaneously targeted *via* one pharmacological inhibitor if possible. Oral administration of several CysLT1 antagonists showed successful improvement in learning behaviors, reduced cell death and proinflammatory cytokines in rat models (Michael et al., 2019). There have been no previous studies directly investigated the use of tomelukast in the AD animal model, so the clinical effects require further discussion.

Tomelukast also targets the peroxisome proliferator-activated receptor (PPAR) *α* and PPARγ in which it regulates carbohydrate and lipid metabolism (Feinstein, 2004). Among the PPARs, PPARγ was reported to exert an anti-inflammatory action upon its activation in the brain; thus, it was suggested that PPARγ agonists could produce protective effects in neurons (Feinstein, 2004). The *in-vivo* model for AD demonstrated that besides the inhibition of T-cell activation and reduction in inflammatory gene expression, PPARγ agonists also increase levels of neuroprotective and anti-inflammatory molecules, including heat shock protein 70 (HSP70) and inhibitory proteins NF-kappa-B inhibitor alpha (IkBα) and NF-kappa-B inhibitor beta (IkBβ) (Feinstein, 2004). Besides the therapeutic benefits tomelukast appears to have as a drug candidate, side effects should be determined to evaluate its long-term use. In rodents, tomelukast are hepatomegaly associated with peroxisome proliferation, and chronic exposure in monkey led to severe diarrhea, anorexia and hypermotility (Hagopian, 1988; Hoover, 1990).

#### Ginkgolide A

Ginkgolide A (GA) is generally considered as a highly active platelet-activating factor antagonist, originally isolated from the leaves of the ginkgo biloba. This herb-derived drug shows therapeutic benefits in inflammatory, cardiovascular and neurological disorders (Koch, 2005). Extensive evidence have suggested that ginkgolide biloba extracts exert various neurobiological effects *via* macromolecular targets on cognitive functions.

One of the macromolecular targets of GA is α-tubulin in microtubules (MT) (Kawamura et al., 2016). In fact, GA was found not to affect MT-assembly, but it selectively modulates the post-translational modifications (PTMs) of α-tubulin, particularly, the detyrosination-tyrosination cycle. Microtubules are composed of tubulin dimers which are subjected to pathological tau-induced PTMs, one of the key hallmarks associated with AD (Kawamura et al., 2016). Decreased level of α-tubulin negatively corresponds to increased polyglutamylation, tyrosination, and detyrosination observed in AD brain (Zhang et al., 2015). Studies have suggested GA could inhibit MT detyrosination and microtubule-organizing centers reorientation, which is vital to the control of neural polarization and axonal specification in AD pathogenesis (Kawamura et al., 2016). GA alleviates microtubule reduction and preserves MT dynamics, further reinforces its possibility in AD drug repurposing.

NMDA receptor antagonists such as memantine have been clinically used to treat mild to severe AD patients. GA as an inhibitor for NMDA, α-amino-3-hydroxy-5-methyl-4-isoxazolepropionic acid (AMPA) receptors as well as other related pathways were thought to be an alternative compound (Kuo et al., 2018). Mechanism of how GA antagonizes these receptors still requires investigation. GA can also alleviate the Aβ-induced pathophysiological status in neurons *via* a decrease in JNK phosphorylation, which has been implicated in the early apoptosis stage (Krilleke et al., 2003; Kuo et al., 2018). Signs of memory improvement by the drug were exhibited in mice models.

**FIGURE 4:**
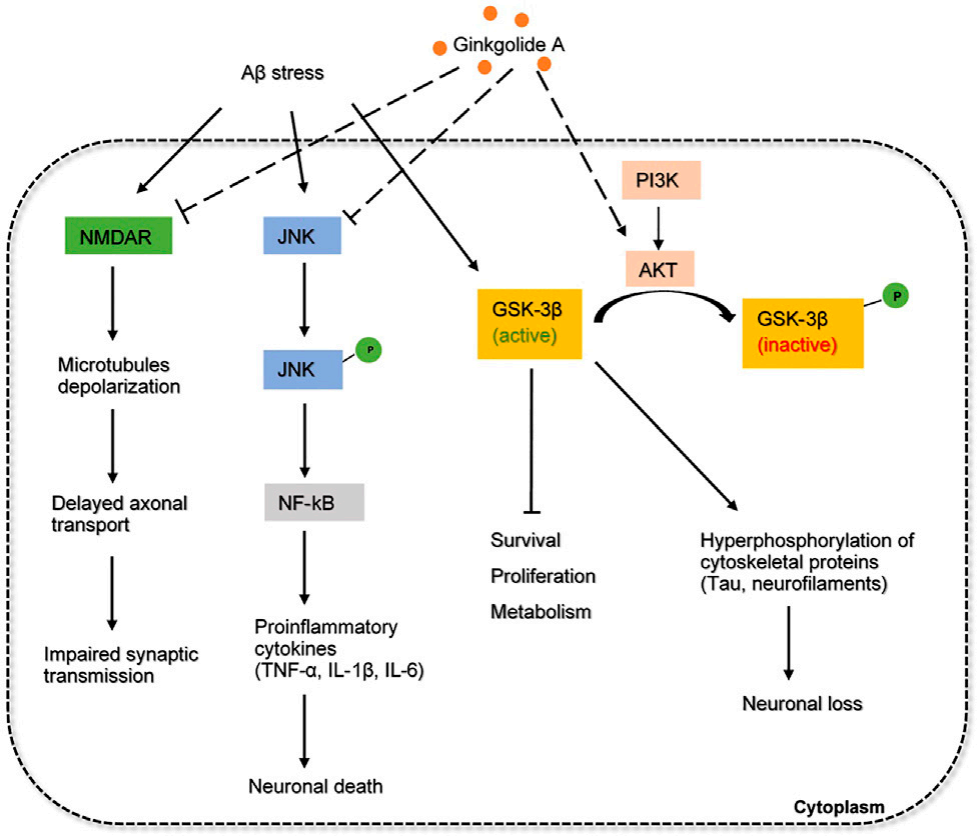
The inferred mechanism of ginkgolide A in AD pathology. The drug acts through the inhibition of NMDA receptor, JNK signaling and the activation of PI3K-AKT pathway. Inhibition of NMDA receptor prevents excessive amounts of glutamate that are associated with synaptic dysfunction and tau phosphorylation. Inhibition of JNK pathway decreases the production of proinflammatory cytokines involved in neuroinflammation. Activation of PI3K-AKT pathway strengthens the inhibition of GSK3β and further prevents the hyperphosphorylation of tau proteins. The solid dash lines show the interactions of protein kinases and signaling cascades. The long-dashed lines show the pharmacological action of the drug.

Another interesting target of GA is phosphoinositide 3 kinase (PI3K)- serine/threonine kinase (Akt) signaling pathway. Akt is a serine-threonine protein kinase downstream of PI3K that can phosphorylate GSK3β at ser9 (Chen et al., 2012). GSK3β-induced tau hyperphosphorylation is one of the main neuropathological events that lead to neurofibrillary degeneration of AD. The underlying mechanism of Akt on the phosphorylation of GSK3β is not clear, but it has been demonstrated that the PI3K-Akt signaling pathway is necessary and sufficient for neuronal survival (Dudek et al., 1997). It was found that GA could increase cell viability and suppress the phosphorylation of Tau by promoting the PI3K-Akt signaling pathway (Chen et al., 2012). Therefore, the regulation between protein kinases and protein phosphatases is well balanced. These results further supported that GA had the potential to improve neuropsychiatric symptoms in the treatment of AD, making GA a potential candidate to prevent Tau accumulation in AD. The overall mechanism of GA involved in AD pathology is proposed and depicted in Figure 4.

Current clinical trials have been undertaken to determine the clinical efficacy of ginkgolides (ginkgolides A, ginkgolides B and ginkgolides C) extracts in the treatment of acute ischemic stroke. However, no related clinical studies on the treatment of AD or memory improvement is found (ClinicalTrials.gov, 2018). Overall, GA would likely be a candidate for drug repurposing for AD, given its multi-targeting effects in various AD pathologies.

#### Chrysin

Chrysin is a naturally occurring flavone commonly found in plants and honey that has proved to modulate a wide range of pharmacological properties with minimum side effects. The drug possesses potent anti-diabetic, anti-inflammatory effects in addition to antioxidant activities (Siddiqui et al., 2018), but it has not been primarily investigated for therapeutic potential in AD.

Chrysin has shown efficacy in attenuating inflammation and neurotoxicity in reactive glial cells. The drug regulates NF-kB p65 and CCAAT/enhancer-binding proteins (C/EBPs) transcription factors where their increased binding to inducible nitric oxide synthase (iNOS) and TNF-α genes could produce a series of neurotrophic compounds (Gresa-Arribas et al., 2010). These compounds mediate the neurotoxic effect of reactive glial cells. Studies have demonstrated that a pre-treatment of chrysin could inhibit NF-kB and C/EBPs protein levels (Gresa-Arribas et al., 2010). Therefore, the inhibition of nitric oxide (NO) and TNF-α production could in turn account for the chrysin’s neuroprotective effect. The presence of the inflammatory response induced by reactive glial cells have been described as a therapeutic interest in AD; thus, the ability of chrysin to downregulate inflammation would make it appear to be a potential drug repurposing candidate.

Due to the poor intestinal absorption, high metabolism and rapid elimination, chrysin could not exert full intrinsic activity. For this reason, Vedagiri et al. (Vedagiri and Thangarajan, 2016) embedded chrysin within lipid nanoparticles (SLNs) in animal models, so the loaded SLNs were attained a longer circulation time in the system. It was found the coated chrysin could improve memory function in the hippocampus caused by Aβ deposition, and restore the level of antioxidant enzymes. Among them, the activity of superoxide dismutase (SOD) and catalase (CAT) was found to be significantly restored in the hippocampus. More importantly, less sign of neuronal degeneration and condensation was observed in rat brain. Chrysin has been shown to induce toxicity in rat liver cells, but low doses of the compound generally have minimum side effects (Siddiqui et al., 2018).

**FIGURE 5:**
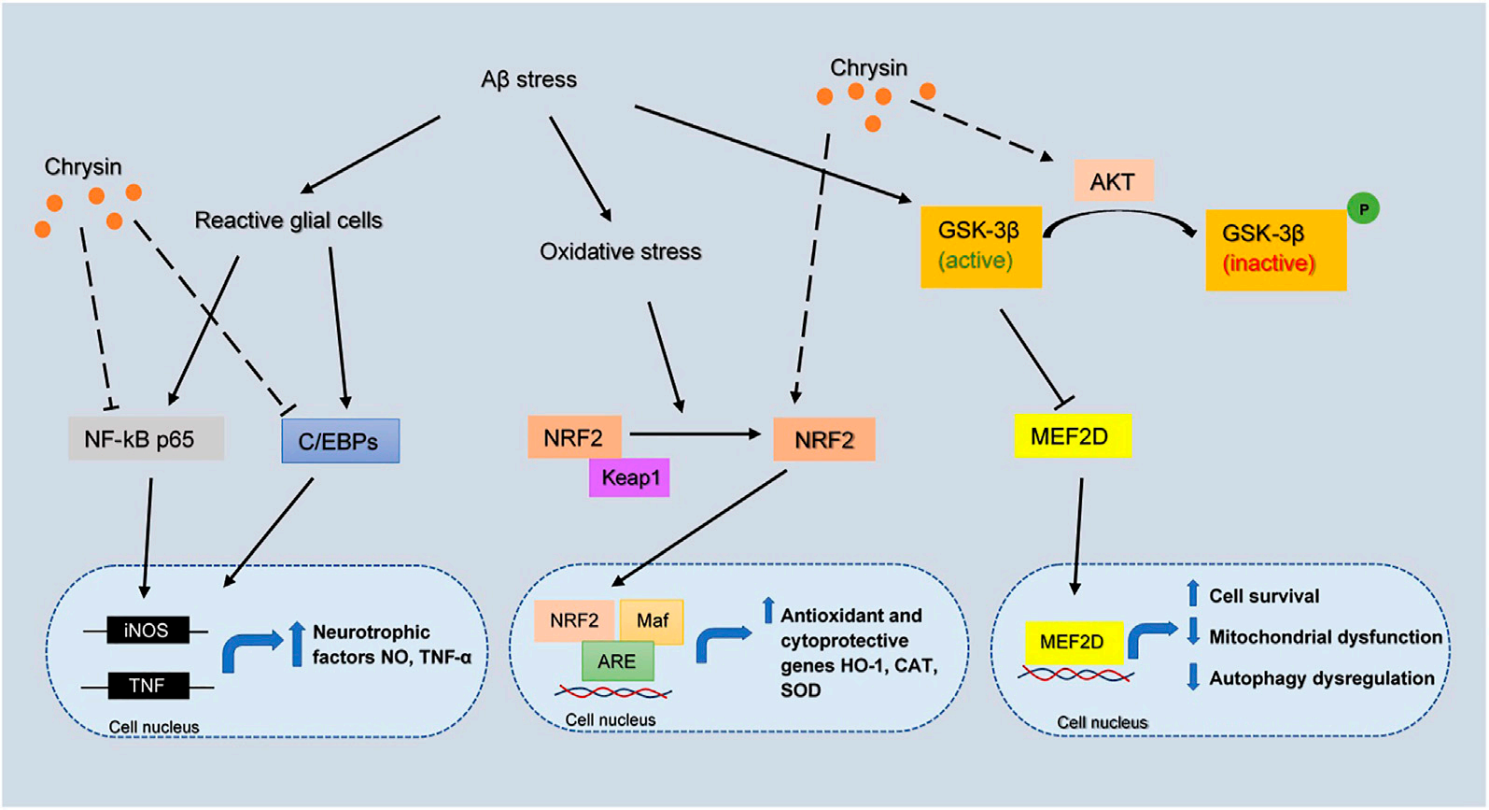
The inferred mechanism of chrysin in AD pathology. The drug acts through inhibition of NF-kB p65, C/EBPs and activation of NRF2, AKT pathway. The inhibition of NF-kB p65 and C/EBPs leads to decreased transcription of iNOS and TNF genes which contributes to the production of neurotrophic factors. The activation of NRF2 leads to the binding with Maf and ARE which increases the transcription of antioxidant and cytoprotective genes HO-1, CAT, and SOD. The strengthening of AKT pathway prevents the deactivation of MEF2D by GSK3β, where MEF2D is a key regulator for cell survival, mitochondrial dysfunction and autophagy dysregulation. The solid lines show the interactions of protein kinases and translocation into the nucleus. The long-dashed lines show the pharmacological action of the drug.

Chrysin has been demonstrated to exhibit antioxidative effects to dopaminergic neurons mainly by increasing the expression of nuclear factor erythroid 2-related factor 2 (NRF2) under oxidative stress (Angelopoulou et al., 2020). The activated NRF2 interacts with the antioxidant responsive element (ARE) and MAF bZIP transcription factor (Maf) to upregulate antioxidant and cytoprotective genes, including heme oxygenase-1 (HO-1), CAT and SOD. Chrysin can also act neuroprotectively through the activation of myocyte enhancer factor 2D (MEF2D) by phosphorylating GSK3β (Angelopoulou et al., 2020). MEF2D is a transcription factor that plays a critical role in dopaminergic neuronal survival in neurological disorders, possibly in AD. In addition, the increased activity of MEF2D can promote cell survival, inhibit mitochondrial dysfunction and autophagy dysregulation (Angelopoulou et al., 2020). Consequently, targeting MEF2D presents a potential therapeutic strategy in AD and chrysin acting as a MEF2D activator could be a useful agent toward this direction. The overall mechanism of chrysin involved in AD pathology is proposed and depicted in Figure 5.

#### Ouabain

Ouabain is a cardiotonic steroid hormone present in both plants and mammals, it also plays a role as a stress hormone synthesized from adrenal cortex (Cavalcante-Silva et al., 2017). Ouabain is a Na+/K+ - ATPase (sodium pump) inhibitor that exerts pharmacological effects on cardiovascular disease and blood pressure control. Na+/K+ - ATPase mediated signaling pathway can induce downstream cascades which regulate the cellular process, such as cell proliferation, hypertrophy and apoptosis (Cavalcante-Silva et al., 2017). Additionally, ouabain was demonstrated to modulate various immune system functions, including inflammation by binding with the Na+/K+ pump in a dose-dependent manner. At low concentrations, this steroid will trigger Na+/K+ pump and cause pro-inflammatory effect by upregulating NF-kB, nucleotide-binding domain-like receptor protein 3 (NLRP3), interleukin one beta (IL-1β). The activated sodium pump will elevate Ca 2+, which eventually results in neuronal injury and cognitive impairment (de Lores Arnaiz and Ordieres, 2014). Inversely, the anti-inflammatory effect of ouabain in inhibiting Na+/K+ pump from reducing NO and IL-1β levels, as well as NF-kB translocation, was observed in rat hippocampus. Overall, the interaction between neuroinflammation and ouabain needs to be more substantially elaborated.

**FIGURE 6:**
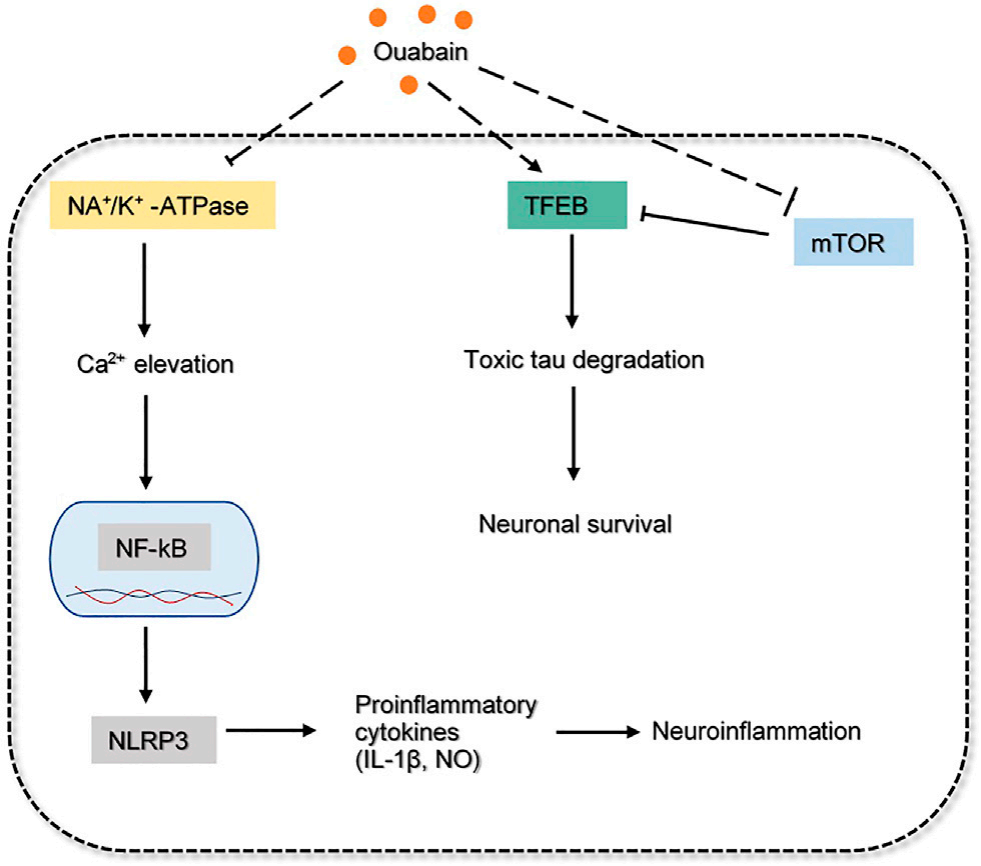
The inferred mechanism of ouabain in AD pathology. The drug acts through the inhibition of Na/K^+^-ATPase, mTOR and activation of TFEB. The deactivation of Na/K^+^-ATPase prevents excessive Ca^2+^ influx that leads to production of proinflammatory cytokines. The drug inhibits mTOR while augmenting TFEB can indicate a better efficacy in toxic tau degradation leading to neuronal survival. The solid dash lines show the interactions of protein kinases. The long-dashed lines show the pharmacological action of the drug.

The accumulation of toxic phosphorylated tau proteins is generally degraded through fusion with lysosomes. In fact, dysregulation of the autophagy system is reported in AD models, suggesting that strengthening the autophagy-lysosome system may garner interest as an alternative therapeutic target (Song et al., 2019). Ouabain has been shown to target transcription factor EB (TFEB), the master regulator of the autophagy-lysosome system. Studies have shown that the enhancement of TFEB increases lysosomal degradative pathways, as a result, it induces APP degradation in AD mice (Xiao et al., 2015). It was revealed that ouabain further contributes to the activation of TFEB by inhibiting the mammalian target of rapamycin (mTOR) in neuronal cells and promoting binding of calcineurin to Na+/K+ - ATPase *in vivo* (Song et al., 2019). The overall mechanism of ouabain involved in AD pathology is proposed and depicted in Figure 6. Overall, this evidence suggested the anti-inflammatory and neuroprotective effect of ouabain in AD pathology, but it might require further investigation on the dose-dependent pathway. Its moderate toxicity profiles also makes it possible for clinical investigation.

#### Sulindac Sulfide

Sulindac sulfide is a non-steroidal anti-inflammatory (NSAID) drug commonly used to treat pain and inflammation (Steinbrink et al., 2009). The drug has also been investigated in the treatment of AD and carcinoma since it targets some proteins with regards to neuroinflammation, amyloidogenesis and cell growth (Wick et al., 2002; Lee et al., 2008).

One of the molecular targets of sulindac sulfide is cyclooxygenase (COX) enzyme, and the overexpression of COX-2 can accelerate neuronal aging and memory deficits. It has been shown that the broad effects of COX inhibition through the long-term treatment of NSAIDs could prevent the early accumulation of Aβ oligomers, and cognitive deficits (Woodling et al., 2016). Results from pretreatment of sulindac sulfide are consistent by showing reduced amyloidogenesis, as well as the Aβ-induced neuroinflammation in mice (Lee et al., 2008). Notably, sulindac sulfide is also a noncompetitive inhibitor for γ42-secretase *in vitro* that preferentially reduces Aβ42 generation, which partially alleviates neurotoxicity and memory impairment in AD (Takahashi et al., 2003). In addition to the regulation of Aβ aggregation by inhibiting γ-secretase, sulindac sulfide could also interfere with existing amyloid plaques, which are the main features in AD. It was shown that in the presence of sulindac sulfide, the precipitation of Aβ was accelerated into prefibrillar aggregates and less toxic higher molecular oligomers. Small soluble oligomers are significantly more toxic and more active on neuronal cells; thus, there is a possibility that the drug could help reduce neurotoxicity by regulating biochemical equilibrium (Yang et al., 2016).

Sulindac sulfide is a defined PPARγ agonist that can improve cognitive function impaired by oxidative stress, neuroinflammation and other related cellular pathways in AD (Khan et al., 2019). PPARγ directly binds to the NF-kB promoters to prevent the production of various proinflammatory cytokines; thus the activation of microglia can be ameliorated. PPARγ exhibits pleiotropic physiological functions in multiple systems; for instance, it can also elicit a reduction in amyloid pathology through ubiquinone mediated degradation of APP (Khan et al., 2019). Overall, augmenting PPARγ expressions by using agonists might be a novel approach for treating AD, sulindac sulfide targets PPARγ makes it possible for drug repurposing. Although two FDA-approved PPARγ agonists (rosiglitazone, pioglitazone) have been tested for their efficacy in mild AD patients to show enhanced memory, sulindac sulfide has not been clinically investigated as a candidate for AD treatment (Landreth et al., 2008). Currently, sulindac sulfide is primarily used as an anti-cancer drug, and it presents gastrointestinal effect including ulceration, bleeding and perforation (Park, 1982).

#### Lorglumide

Lorglumide is a cholecystokinin (CCK) antagonist that inhibits gastrointestinal motility and reduces gastric secretion. It targets both CCKA) and CCKB receptors with a higher selectivity for CCKA subtype (Gonzalez-Puga et al., 2005). Lorglumide has been suggested as a treatment for a variety of gastrointestinal diseases such as pancreatic disorder, irritable bowel syndrome and dyspepsia, and some forms of cancer (Niculescu et al., 2019). Although animal and human studies have produced consistent therapeutic benefits in these physiological disorders, the involvement of CCKA in brain functioning is often unappreciated. CCK is a gastrin-like peptide widely distributed in the gastrointestinal tract and mammalian brain that binds to the CCKA and CCKB receptors (Ballaz, 2017). Malfunction of CCK results in unstable weight regulation and malnutrition seen in 40% of patients with AD, suggesting a link to decreased satiety hormones or decreased sensitivity to these hormones (Plagman et al., 2019). CCKA receptor is a primary physiologic mediator of pancreatic enzyme secretion, and it also fulfills essential functions in the brain, such as the facilitation of dopamine neurotransmission in mesolimbic pathways (Ballaz, 2017). Therefore, the overactivity of CCKA is likely to contribute to the development of neurological disorders by producing an increased dopamine release level. Existing data suggested that dopamine levels were higher in the hippocampus and cortex of the AD patients (Pan et al., 2019), so inhibition of CCKA receptor-mediated activity might provide insights for treating symptoms of AD. Contrarily, recent studies demonstrated that higher CCK levels are also related to a decreased likelihood of having a mild cognitive impairment and AD symptoms (Pan et al., 2019). Meanwhile, the memory-enhancing activity of enterostatin was seen inhibited by pretreatment with lorglumide in the rat model (Ohinata et al., 2007). Due to the inconsistent animal model outcomes, the role of lorglumide requires more preclinical evidence to determine whether there is a neuroprotective or neurotoxic effect in AD pathologies. In terms of side effects, lorglumide is relatively low toxicity and is also active after oral administration (Makovec et al., 1987).

## Discussion

The high-quality genomic data from the single-cell transcriptomic study and GWAS study for AD were taken advantage of to determine potential candidate drugs that could be repurposed or developed. The genomic data sets selected for bioinformatic analysis (see Methods) contains major known AD-associated genes (BIN1, ABCA7, APOE, CLU, and PICALM). These genes were tightly linked to late-onset AD by playing essential roles in APP catabolism according to the Enrichr pathway analysis. Beyond the recognized AD genes identified in the gene lists garnered from the GWAS and single-cell RNA-seq studies, several other genes (TREM2, CD33, CHRNA2, PRSS8, ACE, TKT, APP, and GABRA1) were found to be the targets of clinical AD drugs reported by Cummings et al. (2019). It is believed that these gene lists are necessary for AD drug prediction given the possibility of potential AD drug targets. As a result, eight candidate drugs were obtained from the repurposing analysis. These drugs are involved in the common biological pathways with the 972 prioritized risk genes by combining GWAS and single-cell transcriptomic studies. A literature validation was further conducted to check whether these drugs are involved in clinical trials or animal studies, and possible adverse effects were also taken into account. Some promising drug candidates are ginkgolide A, alsterpaullone, chrysin and ouabain, due to the evidence that they are involved in multiple AD subpathologies in animal models. Only basic neurological research has been done on these drugs, and no clinical trials have been conducted to investigate the impact of these drugs in improving AD symptoms.

Despite enormous effort, the pathophysiology of AD is still not fully understood. The only clinically used and FDA-approved drugs for AD are acetylcholinesterase inhibitors that aim to increase neurotransmission, but they only mildly relieve symptoms. Therefore, developing therapies targeting other subpathologies in AD, such as neurofibrillary tangles, neuroinflammation, oxidative stress, and many others, could be beneficial. For this reason, using drugs that has multiple targets may act on several AD-relevant pathways/targets simultaneously. Repurposing for multi-target compound may be superior to other combination therapies owning to the potential lower risk of drug-drug interaction, but it stills requires further validation. GA was found to be a neuroprotective multi-target drug that is involved in the neuronal transmission, neuroinflammation and toxic protein aggregates. The simultaneous engagement of multiple targets could result in synergistic therapeutic effects, making GA suitable for repurposing in AD research. There have been other drug repurposing studies performed in the past that also indicated the potential of GA. Niculescu et al. (2019) used blood gene expression biomarkers for AD to identify candidate drugs through drug repurposing analysis, and GA was reported with neuroprotective effects. Similarly, alsterpaullone has multiple targets, and it can intervene with the presymptomatic stage and disease progression stage, and its low toxicity profile makes it possible for clinical trials. Chrysin has very mild side effects; currently, it is under basic research in inflammation and neurological disorders (Rashid and Sultana, 2015). The anti-inflammatory effect and symptomatic improvement associated with this drug make it compatible with redesigning and repurposing.

There are many other AD drug repurposing studies performed in the past that share similarity but are ultimately different from the one we conducted. Kwok et al. (2018) used large-scale data and bioinformatics tools to obtain genes strongly associated with AD from the SNP-based GWAS study. Using the late-onset AD genes, they further classified them into clusters based on the encoding protein families. AD drugs are then identified using gene-to-drug cross-references. In contrast, our study had a much more comprehensive set of data to work with: 255 AD genes from Jansen et al. (2019) (71,880 cases, 383,378 controls) and 717 cell-type-specific risk genes from the Mathys et al. (2019) (80,660 single-nucleus transcriptomes), while Kwok et al. (Kwok et al., 2018) only obtained nine gene cluster for drug repurposing analysis which explains why the study provided no evidence of approved or investigational drugs. Our study was also conducted using a novel approach that could prioritize drugs that directly target the AD risk genes and indirectly modulate the target-related pathways. This gave us a more confident drug prediction result as opposed to manually searching for drug candidates using gene-to-drug databases.

Another AD drug repurposing study performed by Siavelis et al. (2015) used gene expression data from five disease-related microarray data sets of hippocampal origin. In contrast to our analysis, gene expression levels were looked at through scRNA sequencing data to find cell-type-specific genes with significantly altered expression in AD patients. Instead of gene sets from the hippocampal region, our study integrated cell-type-specific gene sets from the prefrontal cortex during early and late AD pathology, future analysis could take both areas of brain tissues into consideration for a more robust analysis of transcriptional alterations associated with AD progression. In terms of methodologies, Siavelis et al. (Siavelis et al., 2015) used a more integrative approach with three different methods of evaluating differential gene expression and four drug repurposing tools. They obtained a list of 27 potential anti-AD compounds that were further processed with pathway enrichment and network analysis. However, our study only used one drug repurposing tool and the drug list was validated with literature mining.

Few studies have used the AD genomic data obtained from both GWAS and scRNA-seq for clinical purposes, making this sort of drug repurposing study useful. Development of AD drugs is highly desirable given the lack of efficacy over time of current AD symptomatic drugs. The drug repurposing method makes use of large-scale genomic data and alleviates the cost associated with drug development. This approach advances our understanding of AD pathology by targeting pathways directly or indirectly interact with risk genes, and it further facilitates personalized medicine based on the patient’s genomic composition and AD progression stage.

There are several gaps in the method approach and literature review that can be identified from the current study. There were six major cell types of scRNA-seq data identified from Mathys et al. (2019), but only gene sets from three major cell types (excitatory neurons, microglia, oligodendrocytes) were selected for drug repurposing analysis. We will consider incorporating more cell types and other AD-specific scRNA-seq studies in the future research to revise our framework. Meanwhile, the function of these risk genes and genes targeted by candidate drugs needs further investigation to identify more promising drugs. The lack of convincing literature evidence regarding drug targets and the controversial biology of drug-induced pathways led to the exclusion of several drugs such as lorglumide and ellipticine. Besides, severe side effects associated with some candidate drugs, including tomelukast and sulindac sulfide, prohibited their potential in clinical studies and long-term safety.

## Conclusion

In this study, we applied a computational approach Gene2Drug on the large-scale genomic data for rational AD drug repurposing integrating drug-induced transcriptional responses with the annotated pathways databases. This method is complementary to other leading computational tools which exploits gene-protein interaction networks (Napolitano et al., 2017). The semi-automated method allows more flexibility in choosing pathways that better describe the functions of risk genes, prioritizing drugs deemed therapeutic. Although Gene2Drug has been shown effective at identifying drugs with desired effect, any unrelated drugs indirectly modulate the expression of target-drug pathways will also be selected, which requires a more rigorous screening process on the candidate drugs. The method can be easily extended to larger databases, such as Library of integrated network-based cellular signatures (LINCS) databases, in this case, the method may be significantly improved by including more pathways analysis. In conclusion, we prioritized four potential candidate drugs with repurposing potential for AD. Remaining work on the experimental validation of these candidates is required, which will be aided by the continuously increasing information on genes, drugs, and the proteins they target.

## Data Availability

Data have been downloaded from https://www.nature.com/articles/s41588-018-0311-9#Sec28; https://www.nature.com/articles/s41586-019-1195-2.
